# How often should dead-reckoned animal movement paths be corrected for drift?

**DOI:** 10.1186/s40317-021-00265-9

**Published:** 2021-10-16

**Authors:** Richard M. Gunner, Mark D. Holton, David M. Scantlebury, Phil Hopkins, Emily L. C. Shepard, Adam J. Fell, Baptiste Garde, Flavio Quintana, Agustina Gómez-Laich, Ken Yoda, Takashi Yamamoto, Holly English, Sam Ferreira, Danny Govender, Pauli Viljoen, Angela Bruns, O. Louis van Schalkwyk, Nik C. Cole, Vikash Tatayah, Luca Börger, James Redcliffe, Stephen H. Bell, Nikki J. Marks, Nigel C. Bennett, Mariano H. Tonini, Hannah J. Williams, Carlos M. Duarte, Martin C. van Rooyen, Mads F. Bertelsen, Craig J. Tambling, Rory P. Wilson

**Affiliations:** 1 Swansea Lab for Animal Movement, Department of Biosciences, Swansea University, Singleton Park, Swansea SA2 8PP, Wales, UK; 2 School of Biological Sciences, Queen’s University Belfast, Belfast, 19 Chlorine Gardens, Belfast BT9 5DL, Northern Ireland, UK; 3 Biological and Environmental Sciences, University of Stirling, Stirling FK9 4LA, Scotland, UK; 4 Instituto de Biología de Organismos Marinos (IBIOMAR), CONICET. Boulevard Brown, 2915, U9120ACD Puerto Madryn, Chubut, Argentina; 5 Departamento de Ecología, Genética y Evolución & Instituto de Ecología, Genética Y Evolución de Buenos Aires (IEGEBA), CONICET, Pabellón II Ciudad Universitaria, C1428EGA Buenos Aires, Argentina; 6 Graduate School of Environmental Studies, Nagoya University, Furo-cho, Chikusa-ku, Nagoya, Japan; 7 Organization for the Strategic Coordination of Research and Intellectual Properties, Meiji University, Nakano, Tokyo, Japan; 8 School of Biology and Environmental Science, University College Dublin, Belfield, Dublin, Ireland; 9 Savanna and Grassland Research Unit, Scientific Services Skukuza, South African National Parks, Kruger National Park, Skukuza 1350, South Africa; 10 Veterinary Wildlife Services, South African National Parks, 97 Memorial Road, Old Testing Grounds, Kimberley 8301, South Africa; 11 Department of Agriculture, Government of South Africa, Land Reform and Rural Development, Pretoria 001, South Africa; 12 Department of Migration, Max Planck Institute of Animal Behavior, 78315 Radolfzell, Germany; 13 Department of Veterinary Tropical Diseases, Faculty of Veterinary Science, University of Pretoria, Onderstepoort 0110, South Africa; 14 Durrell Wildlife Conservation Trust, Les Augrès Manor, Channel Islands, Trinity JE3 5BP, Jersey, UK; 15 Mauritian Wildlife Foundation, Grannum Road, Indian Ocean, Vacoas, Mauritius; 16 Centre for Biomathematics, Swansea University, Swansea SA2 8PP, UK; 17 Mammal Research Institute. Department of Zoology and Entomology, University of Pretoria, Pretoria 002., South Africa; 18 Instituto Andino Patagónico de Tecnologías Biológicas y Geoambientales, Grupo GEA, IPATEC-UNCO-CONICET, San Carlos de Bariloche, Río Negro, Argentina; 19 Red Sea Research Centre, King Abdullah University of Science and Technology, Thuwal 23955, Saudi Arabia; 20 Center for Zoo and Wild Animal Health, Copenhagen Zoo, Roskildevej 38, DK-2000 Frederiksberg, Denmark; 21 Department of Zoology and Entomology, University of Fort Hare, Alice Campus, Ring Road, Alice 5700, South Africa

**Keywords:** Biologging, Dead-reckoning, Drift, Global Positioning System (GPS), Animal movement, Animal tracking, Tilt-compensated compass, GPS correction

## Abstract

**Background:**

Understanding what animals do in time and space is important for a range of ecological questions, however accurate estimates of how animals use space is challenging. Within the use of animal-attached tags, radio telemetry (including the Global Positioning System, ‘GPS’) is typically used to verify an animal’s location periodically. Straight lines are typically drawn between these ‘Verified Positions’ (‘VPs’) so the interpolation of space-use is limited by the temporal and spatial resolution of the system’s measurement. As such, parameters such as route-taken and distance travelled can be poorly represented when using VP systems alone. Dead-reckoning has been suggested as a technique to improve the accuracy and resolution of reconstructed movement paths, whilst maximising battery life of VP systems. This typically involves deriving travel vectors from motion sensor systems and periodically correcting path dimensions for drift with simultaneously deployed VP systems. How often paths should be corrected for drift, however, has remained unclear.

**Methods and results:**

Here, we review the utility of dead-reckoning across four contrasting model species using different forms of locomotion (the African lion *Panthera leo*, the red-tailed tropicbird *Phaethon rubricauda*, the Magellanic penguin *Spheniscus magellanicus*, and the imperial cormorant *Leucocarbo atriceps*). Simulations were performed to examine the extent of dead-reckoning error, relative to VPs, as a function of Verified Position correction (VP correction) rate and the effect of this on estimates of distance moved. Dead-reckoning error was greatest for animals travelling within air and water. We demonstrate how sources of measurement error can arise within VP-corrected dead-reckoned tracks and propose advancements to this procedure to maximise dead-reckoning accuracy.

**Conclusions:**

We review the utility of VP-corrected dead-reckoning according to movement type and consider a range of ecological questions that would benefit from dead-reckoning, primarily concerning animal–barrier interactions and foraging strategies.

## Background

Much of animal behaviour is defined by movement patterns in environmental space [1–3]. Today, most researchers use transmission telemetry (e.g., VHF, GPS, and acoustic transmitters) to verify an animal’s location periodically connecting these ‘Verified Positions’ (VPs) linearly in time to reconstruct movement paths [4–7]. Using this approach, researchers can use the combination of step lengths and turn angles as indicative of behaviour, functional motivation, habitat quality, resource selection and networks of space-use [8–12]. While it is acknowledged that more positional fixes enhance our ability to define these metrics, approaches for interpolating space-use depend on the temporal and spatial resolution of the system’s measurement so that obtaining fine-scale, continuous and accurate estimates of animal space-use is not straight forward [13–16]. Specifically, the resolution of tortuosity—how convoluted an animal track is—in animal movement paths is compromised when consecutive VPs are temporally far apart [14, 17], whilst all VP systems are subject to positional inaccuracy [18–20], which can lead to varying assessments of movement [21–23].

GPS units are one of the most technologically advanced (and arguably the most popular) VP systems (cf. [24–26]), capable of recording with high frequency (e.g., 5 Hz) [27] and for many months (although not both simultaneously for reasons of power draw) [28]. Yet, even with GPS, fix success rate can drop dramatically and locational accuracy can easily vary by a few metres or more, depending on the propagation of signal quality and/or receiver reception capability [29, 30]. In addition, these units can be subject to latency delays by up to ~ 5 s [31, 32], whilst most commercial loggers are only precise to around 1 m [22] and so, irrespective of fix accuracy, time-based positional error can accumulate (as a function of sampling rate) when the spatial resolution of animal movement is less than the precision error radius between consecutive readings.

Motion sensor systems (also called Inertial Measurement Units—IMUs) incorporating tri-axial accelerometers and magnetometers are increasingly being used in animal-attached tags to determine fine-scale (second to infra-second) movement of animals via dead-reckoning [33, 34], thereby allowing elucidation of movement-related behaviours (cf. [35–40]). Dead-reckoning involves sequentially integrating travel vectors (heading and speed estimates), radially in time [41] (and 3-D space with aligned pressure/depth data). Compared to animal-borne video recorders and GPS units, motion sensors require far less current (cf. [42, 43]) and operate at much higher recording frequencies and precision [36, 40, 44, 45]. Indeed, studies are increasingly demonstrating the value of motion sensors for resolving continuous and fine-scale movements in 2- or 3-D space, on/in terrestrial—(e.g., [42, 46]), marine (e.g., [47, 48]) and aerial (e.g., [49]) environments—far beyond what would have been obtained using VP systems alone (cf. [14, 24, 46]).

Crucially, inertial measurements, being unaffected by factors that modulate VP accuracy, provide an independent and higher resolution comparator for assessing the extent (and type) of movement undertaken [50], which can be localised in environmental space when paired with a VP system. Uncorrected dead-reckoned paths have been referred to as ‘pseudo-tracks’ [41, 51], because extrapolated travel vectors always incur some error, and being additive [42], even small errors accumulate to have more substantial influences on path shape (conventionally termed ’drift’ [33, 46, 52]). This means that, although the form of animal movement is maintained most accurately by adjacent track sections (e.g., [47, 53]), the relationship between animal path and the environment tends to deviate over time [54]. VPs obtained from a secondary source (e.g., GPS) can correct for this by periodically resetting accumulated drift [41, 46]. Gunner et al. [50] provided a recent reappraisal of the dead-reckoning method in R and noted that the extent of system error that governs dead-reckoning accuracy can be appreciable and should be primarily modulated according to both the species in question (type of movement medium and movement scales) and the VP correction rate used (itself, usually constrained by power consumption). How often one should correct for drift with VPs, however, remains unexplored on wild animals.

Dewhirst et al. [46] were the first to examine this issue on a domestic species, as they compared various scales of GPS-corrected dead-reckoned tracks, obtained from domestic dogs (*Canis lupus familiaris*). Unsurprisingly, position error (the distance between temporally aligned dead-reckoned and GPS positions) decreased as a function of correction rate and the authors concluded that a correction rate of one fix every five minutes resulted in highly accurate distance moved estimates. For similar results using GPS alone, they noted that it would have required 12 fixes per minute (assuming no GPS error) which would have reduced battery life from months to days. Dead-reckoning thus provides the means to extend battery life (or reduce battery size in any tag deployment, with the attendant benefits for animal wellbeing (cf. [55, 56]) and improve the accuracy and detail of behaviourspecific travelling movements between VPs (e.g., [41, 42, 57]). How this extends to wild animals and beyond the terrestrial movement medium, however, requires further investigation.

This study uses the dead-reckoning protocols and R functions described in Gunner et al. [50] to examine the movement of VP-corrected dead-reckoned animal paths for four wild species (a mammal (lions—*Panthera leo*) and three bird species (penguins—*Spheniscus magellanicus*, cormorants—*Leucocarbo atriceps*, tropicbirds—*Phaethon rubricauda*)), varying across greater than an order of magnitude in mass and travelling in the three major media (land, water and air) using walking, swimming and flying. We sub-sampled the scale of VP correction to examine the trends in net error and compared cumulative distance moved estimates between VP-corrected dead-reckoned tracks and GPS paths alone according to VP correction rate and simulated VP down-sampling, respectively. Our goal was to demonstrate that the traditional trade-off between VP correction rate and dead-reckoning accuracy is more complex than simply ‘the higher the rate, the greater the accuracy’ (though this would be the case if VPs were perfect). Rather, the accuracy of both reconstructed paths is heavily dependent on the animal’s lifestyle (including the specifics of location and the speeds and mode(s) of movement). Accordingly, we assess the importance of appropriate dead-reckoned track-scaling, which is primarily based on speed estimates prior to VP correction. We also highlight the benefit of acquiring external air-/tidal-flow vectors for animals traveling in fluid media (air and water), particularly when VPs are temporally widely spaced, and emphasise the danger of carrying out VP correction too frequently (irrespective of battery consumption) due to VP error. Lastly, in context of the above, we outline the utility of dead-reckoning across various scenarios and the key considerations for deciding VP correction frequency.

## Materials and methods

### Study species

We selected four free-living species, exemplifying almost two orders of magnitude of mass, which travel in three media. These were; 10 lions (mass *ca*. 130–190 kg; ‘four-legged walkers’), 15 penguins (mass *ca*. 4 kg; ‘two-legged walkers’ and ‘swimmers’), 15 cormorants (mass *ca*. 1.8–3.5 kg; ‘flyers’ and ‘swimmers’) and seven tropicbirds (mass *ca*. 0.6 kg; ‘flyers’). Animals were equipped with Daily Diaries (DDs) [Wildbyte Technologies—http://www.wildbytetechnologies.com/], recording tri-axial acceleration, magnetic-field intensity and pressure (either barometric- or hydrostatic pressure) [36]. Unencapsulated DD models ranged between 27 × 26 × 10 mm and 26 × 17 × 5 mm and weighed 2–3 g (incl. microSD card and excl. batteries). In tandem, animals were also equipped with GPS (Axytrek or Gipsy) units [https://www.technosmart.eu/], programmed to record at one fix every minute for tropicbirds and one fix every second for the other species. Both encapsulated devices (incl. batteries) always comprised < 3% of the average body mass of each species. Animals were left to roam freely, for periods ranging between 1 to 16 days before the devices were recovered (Table 1).

### VP-corrected dead-reckoning procedure

Tracks were reconstructed and drift corrected using the *Gundogs.Tracks()* function in R (see Gunner et al. [50]), based on the protocols outlined in Walker et al. [41]. See Additional file 1 for the VP correct dead-reckoning formulae. Pitch and roll {representing posture, expressed using Euler angles (cf. [58])}, were calculated from static acceleration estimates [59] and heading was derived using the tilt-compensated compass method [60], with any required magnetic declination offset applied. Pitch (used in the computation of speed for diving animals; see Table 1) and heading were post-smoothed by 1–2 s (i.e., a rolling ‘circular’ mean used for heading values [61]).

We used the Vector of the Dynamic Body Acceleration (VeDBA; see Eq. 1) [62, 63] (smoothed by 2 s) as a speed proxy for terrestrial locomotion [64]:

(1) $$\text{VeDBA}=\sqrt{\left(D_{x}^{2}+D_{y}^{2}+D_{z}^{2}\right)}$$

where *D_x_*, *D_y_* & *D_z_* are the dynamic acceleration values from each axis. The ‘linear’ VeDBA–speed relationships [speed = (VeDBA · m) + c] were derived either by iteratively changing the *m* coefficient (gradient) per individual until (uncorrected) dead-reckoned tracks were scaled according to the corresponding GPS tracks (using a zero *c* constant (intercept)), or by substituting *m*- and c-values with GPS-derived speed *vs* VeDBA regression estimates [23, 46]. For swimming and flying locomotion, where Dynamic Body Acceleration (DBA) is considered a weak proxy of speed (cf. [49, 65]), speed values were allocated according to: (i) behaviour-type (itself, elucidated from motion sensor data (e.g., [39, 66]); (ii) rate of change of depth *versus* dive angle-derived speed [67] or (iii) GPS-derived speed estimates (using the Haversine distance formula [68]) between GPS fixes (at defined fix intervals). Movement-specific behaviours were identified using one or a combination of; visual interpretation of stylised patterns in acceleration data (cf. [37, 69, 70]), the Lowest Common Denominator Method (LoCoD) method [39] and the Movement Verified Filtering (MVF) method [23]. Species’ specific speed allocation details according to movement-specific behaviour and/or topological whereabouts, are given in Table 1. Any required tag orientation offsets (e.g., due to imperfect tag placement along the longitudinal axis of the animal) as well as baseline pressure drift were accounted for by rotation correction of magnetic and acceleration vectors [50, 58] and trend estimation with asymmetric least squares (cf. [71]), respectively.

### VP correction rate and metrics of analysis

All tracks were dead-reckoned at periods between 1 and 10 Hz resolution (Table 1). According to the duration of deployment and GPS fix rate, VP correction rate was thinned at scales of; 1 fix/24 h, 1 fix/12 h, 1 fix/6 h, 1 fix/3 h, 1 fix/1 h, 1 fix/30 min, 1 fix/15 min, 1 fix/5 min, 1 fix/min, 1 fix/30 s, and 1 fix/1 s. Net error and distance moved estimates (in metres) were calculated for each species, individual and VP correction rate. The Haversine distance formula [68] was used to compute 2-D net error, which we define as the distance between every VP (irrespective of VP-correction rate) and the corresponding time-matched VP-corrected dead-reckoned position. Distance moved was summed separately, both between consecutive dead-reckoned positions and consecutive GPS positions, the latter being down-sampled according to the VP correction rate (e.g., if the VP correction rate was approx. 1 fix/h, then GPS data were sub-sampled to this frequency prior to computing distance moved between retained positions). The Haversine formula was used to compute 2-D distance moved (terrestrial ‘on-land’ movement). For 3-D dead-reckoned movements (penguins and cormorants at depth and tropicbirds at altitude), positions were converted to Cartesian coordinates (*x*, *y*, *z*), incorporating the Earth’s oblate spheroid shape (geodetic latitude) and the straight-line distance between sets of Cartesian coordinates were calculated using Pythagorean theorem [50]. 3-D distance moved was not computed between VPs since both the level of VP thinning (particularly at lower VP correction rates) and the periods of time where fix success rate dropped (e.g., under water) made this inappropriate. Altitude (in metres) was calculated using local coastal meteorological recordings of air pressure at 5-min resolution by a portable weather station (Kestrel 5500L, Kestrel instruments, USA) stationed at the highest point above sea-level (*ca*. 280 m) on Round Island, Mauritius [72]. Ocean current vectors were composed from a validated 3-D numerical model constructed for the region [73], with tidal currents deduced hourly at 1-km^2^ resolution.

All VPs (filtering out obvious outliers visually) were used when making inter-specific comparisons of net error estimates across VP correction rates and given that, in this process, VPs are considered the benchmark upon which net error is assessed, net error zeros out when VP correction includes all VPs (VP correction rate = VP logging frequency; see Table 1). In conjunction with the main findings, we report various applications of dead-reckoning and extensions to improve dead-reckoning accuracy, such as the importance of initial speed estimates and incorporation of external current flow vector estimates in fluid media, using various species-specific case-studies as examples.

## Results

**Fig. 1:**
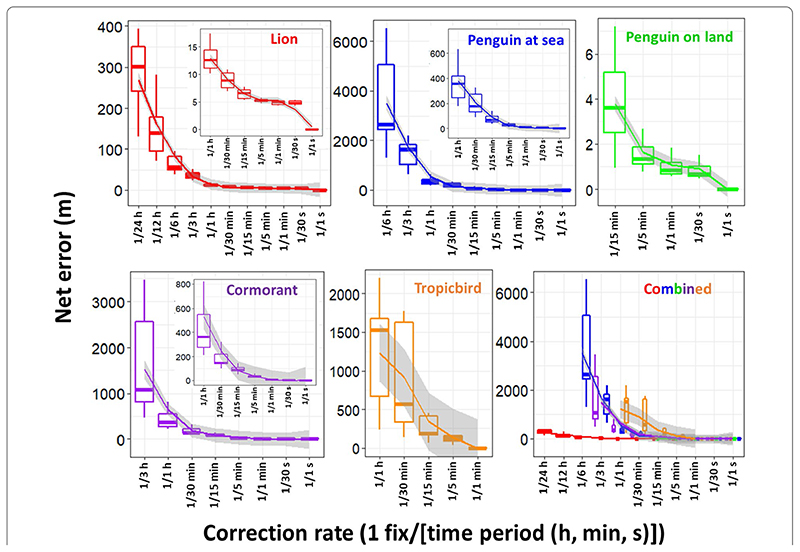
Boxplots summarising the magnitude of net error according to the VP correction rate per species. Mean values were aggregated per individual and VP correction rate. Boxes encompass the 25–75% interquartile range and horizontal bars denote the median value with ‘loess’ smooth line (grey shading shows the standard error and Whiskers extend to 1.5 * Interquartile range). Note net error drops to zero when the VP correction rate equates with GPS recording frequency (1 Hz for the lions, penguins and cormorants, and 1 fix/min for the tropicbirds). The inserts zoom in on the net error between VP correction rates of one fix per hour and one fix per second

Net error decreased with increasing VP correction rate, although the species travelling in fluid media had much larger net error estimates for any given VP correction rate (Fig. 1). For example, considering a VP correction rate of 1 fix/h, the mean net error of penguins (at sea), cormorants and tropicbirds were approximately 28, 42 and 95 times greater, respectively, than lions. A visually obvious ‘plateau’ of net error drop (relative to the initial gradient) varied between species (with respect to magnitude of net error and level of VP correction rate).

**Fig. 2:**
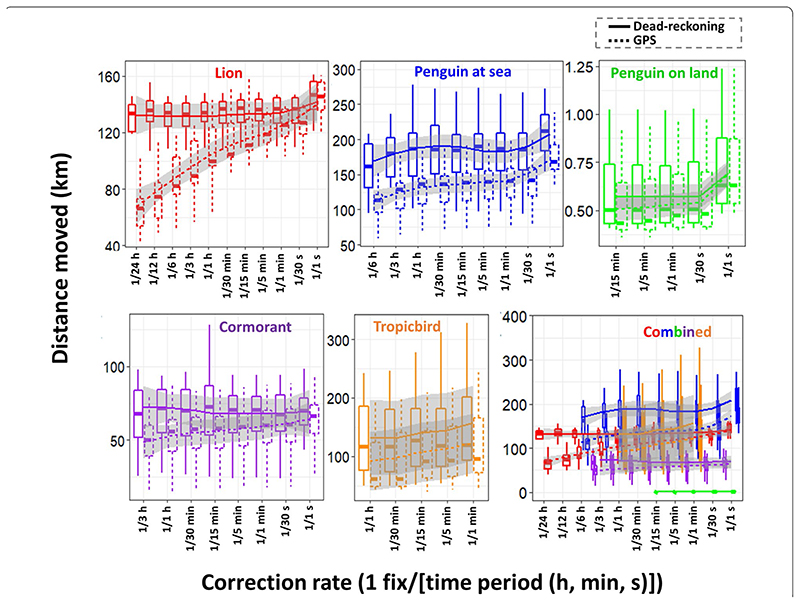
Boxplots demonstrating the total distance moved (km) during the tag deployment period according to VP correction rate for the study species. Solid lines show the distance moved calculated using successive dead-reckoned positions (distance moved (see methods) was 3-D and computed for penguins and cormorants operating at varying depths and tropicbirds at varying altitudes, and 2-D computed for lions and penguins walking on land). Dashed lines reflect the distance moved calculated from successive GPS positions according to the level of VP under-sampling stated (only 2-D distances were computed). Mean values were aggregated per individual and per VP correction rate. Boxes encompass the 25–75% interquartile range and horizontal bars denote the median value with ‘loess’ smooth line (grey shading shows the standard error and Whiskers extend to 1.5 * interquartile range). Note that the high spread of each species boxplot is due to the high intra-specific variability of distances moved—e.g., with the tropicbirds, differences in foraging/distance roamed may be due to breeding vs. non-breeding status

Across all species, estimates of overall distance moved were smaller when summed between GPS positions (thinned according to VP correction rate) than for dead-reckoned positions (Fig. 2). Dead-reckoned estimates of distance moved were generally more consistent across the VP correction rates, relative to the corresponding GPS-derived distance moved estimates (in which VP thinning is equivalent to VP correction rate). Although there are slight variations in the pattern of these trends between species, there was a notable increase when VP correction rate is highest.

**Fig. 3:**
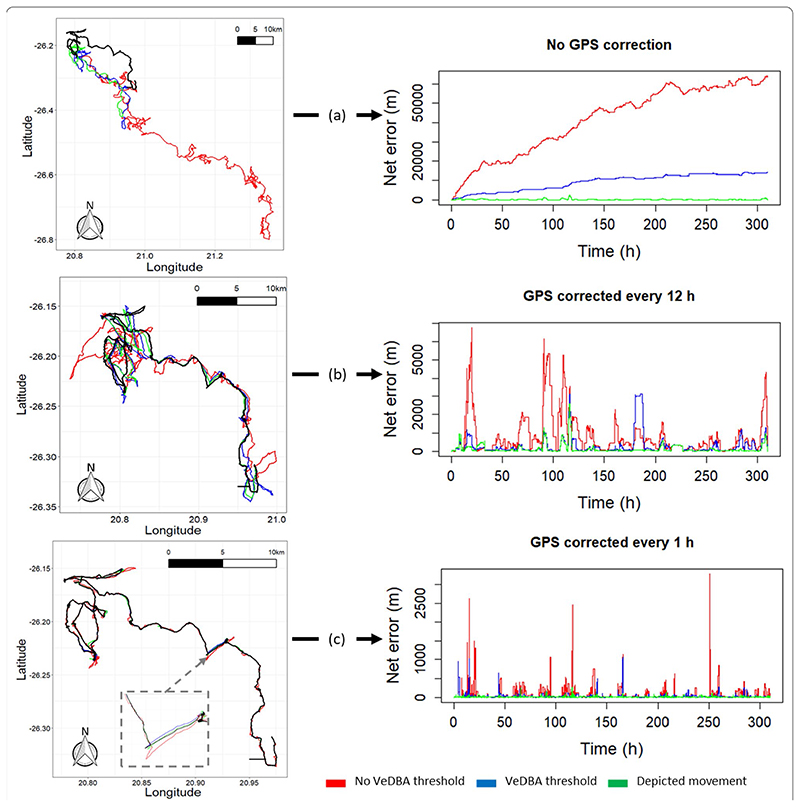
A lion’s dead-reckoned movement path (approx. 12 days) in relation to (all) GPS positions (black), plotted both as a function of GPS correction rate (**a** no correction, **b** GPS corrected every 12 h, **c** GPS corrected once every hour) and initial subset of data used to create the path (red = all data (no VeDBA threshold for speed), blue = only data that surpassed VeDBA threshold (> 0.1 g) used for speed, green = only data during periods depicted as proper movement (using the MVF protocol [23])). Note the difference in y-scales across the net error graphs

Estimates of net error and distance moved were standardised according to the mean time between corrections per VP correction rate and the duration of the movement path, respectively, per individual (Additional file 1: Fig. S1), which further highlighted the trend between speed of movement and net error estimates. Beyond this, the rate of net error was relatively consistent between VP correction rates (Additional file 1: Fig. S1), demonstrating that the ‘plateaus’ of net error drop observed in Fig. 1, are primarily the result of the non-linear scales of VP correction thinning (although there was a noticeable minor decreasing trend from lowest to highest VP correction rates for animals moving in fluid media). Alongside VP-correction rate, dead-reckoning accuracy was heavily affected by the initial scaling of dead-reckoning tracks (Fig. 3). This principally related to appropriate speed allocation, such as threshold values of DBA to estimate speed and only advancing tracks at times of known travelling movements, thus excluding DBA values due to movements which do not lead to a displacement of the body in space (e.g., self-grooming movements). Here, per given VP correction rate, tracks advanced only during times of depicted movement (using the MVF protocol [23]; green) recorded the lowest net error, relative to using all data (red) and subset data using a VeDBA threshold (blue) (Fig. 3). Whilst net error generally did not vary strongly with activity level (of which VeDBA is a proxy) for lions, the variance was markedly higher at both high and low VeDBA values, indicating that correcting for VP error during inactivity may be just as important as the initial track-scaling (Additional file 1: Fig. S2).

**Fig. 4:**
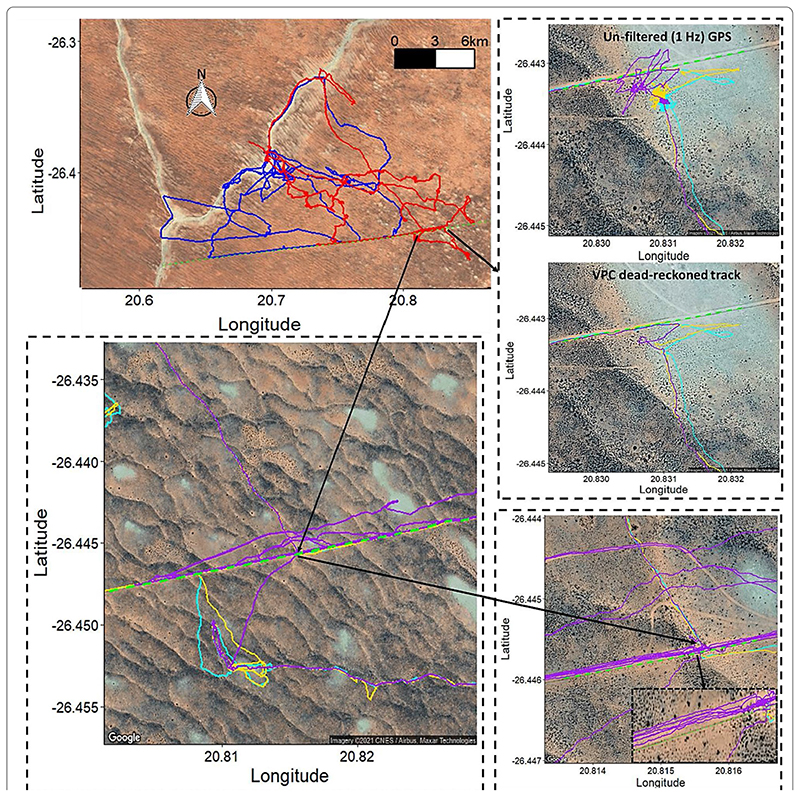
VP-corrected dead-reckoned movements of lions in the Kgalagadi Transfrontier Park. The top left plots show a pride of 5 lions (2 males—blue and 3 females—red). Both male and female movements abutted the Botswana fence boundary (dashed green line), although only the females crossed (illustrated in the dotted inserts, with yellow, cyan and purple tracks denoting individual females) The bottom right insert shows one female pacing along the fence line in an attempt to re-join the other two that crossed hours earlier. Note the extent of (unfiltered) GPS error that occurs (particularly during resting behaviours) (top right)

VP-corrected dead-reckoning provided the means to investigate behavioural responses with higher resolution, without incorporating the inaccuracies of positional noise associated with VPs obtained at high frequency (Fig. 4). For example, here, VP-corrected dead-reckoning (using a VP correction rate of 1 fix/min) shows the various sites at which three female lions crossed the Kgala-gadi Transfrontier Park fence line (into Botswana from South Africa), including patrolling behaviour of one female (purple) that became separated in time and space (Fig. 4).

**Fig. 5:**
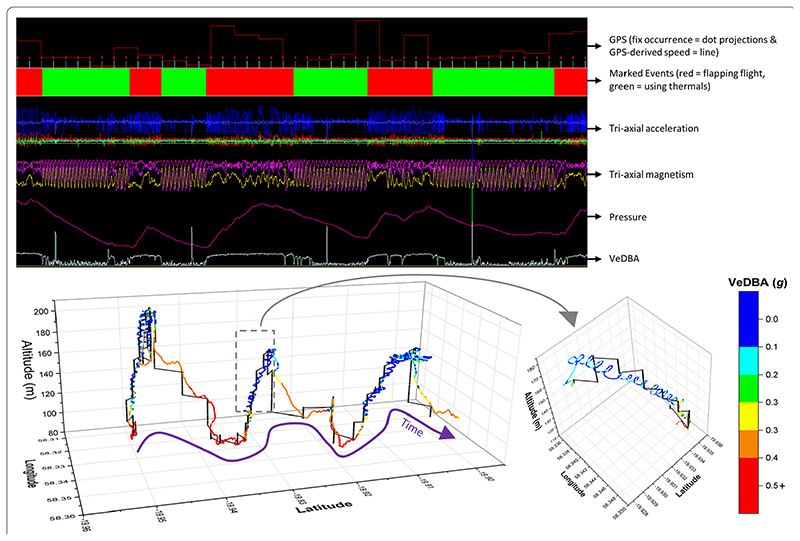
A 45-min section of a tropicbird’s foraging flight at sea, encompassing periods of thermal soaring. The top plot characterises stylised trends in the raw values and select derivatives from the motion sensor and GPS unit outputs (2D waveforms *vs* time), including differentiating flapping flight from thermal soaring (marked events—primarily based on magnetism data). The sine waves appearing in two of the magnetometer channels simultaneously reflect circling. The bottom plot graphs the dead-reckoned track (coloured according to VeDBA) in 3-D, relative to all available GPS fixes obtained (black) (including an insert of circling behaviour). Note periods of thermal soaring are not apparent with GPS at the recording frequency of 1 fix/1 min as used here. Note that climb rate increases as a function of the inverse of the rate of change of pressure

**Fig. 6:**
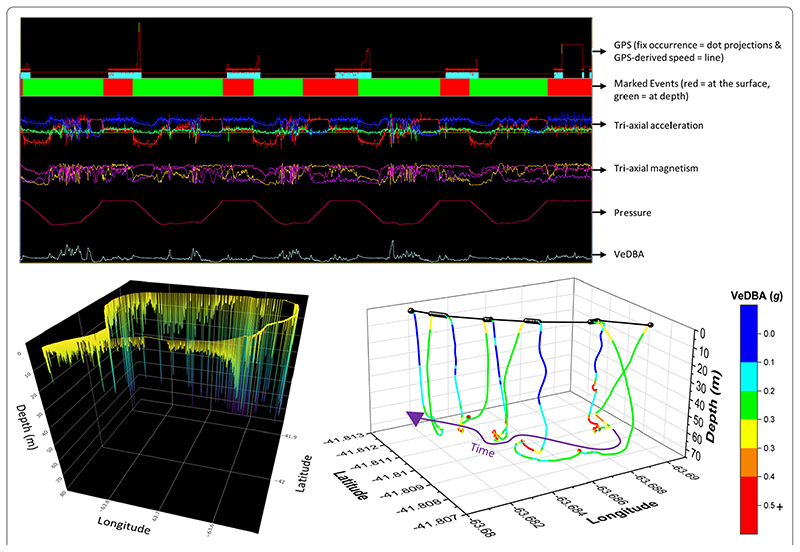
A 15-min duration of a Magellanic penguin’s foraging trip at sea. The top plot characterises stylised trends in the raw values and select derivatives from motion sensor and GPS unit output (2D waveforms vs time), including differentiating between dives and surface periods (marked events—primarily based on depth data). Note that pressure is inverted to reflect depth. The bottom left plot maps the entire (17 h) VP-corrected dead-reckoned foraging trip. The bottom right plot graphs the resultant VP-corrected dead-reckoned track (coloured according to VeDBA) in 3-D, relative to all available GPS fixes obtained (black)). Note the latency delays in GPS recordings (as seen in the top plot), with a temporal offset of fixes (red dot projections) occurring at green-marked events (at depth)). Fixes that occurred at depth were removed from the analysis

Even when dead-reckoned travel vectors likely incorporated appreciable error (e.g., due to low-resolution (constant) speed estimates), fine-scale movement-specific behaviours were apparent (beyond the capacity of the VP systems used), for example, soaring in thermals (Fig. 5) or the tortuosity of foraging (Fig. 6).

**Fig. 7:**
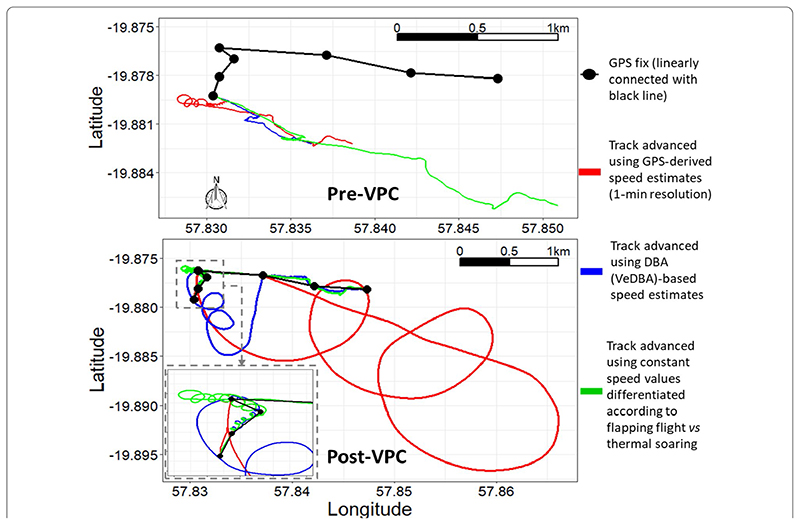
Seven minutes of tropicbird flight with dead-reckoned tracks advanced according to 3 different allocations of speed, plotted alongside GPS (1 fix/min) both pre- and post-VP correction. This demonstrates the main error that can arise during the VP correction procedure (using heading and distance correction factors (see “Discussion” section)), when there is a large disparity in distance between consecutive VPs and consecutive dead-reckoned positions, primarily due to inaccurate speed allocation and/or VP error. Note how a segment of thermalling behaviour was disproportionately expanded during the VP correction process when using GPS-derived speeds and DBA-based estimates, because there was no differentiation between thermal soaring and flapping flight (cf. Fig. 5). Using a much lower speed value during thermal soaring value (a quarter of the magnitude allocated for flapping flight) greatly improved track estimates because the magnitude of linear drift correction works as a function of the underlying speed allocation

We found that the interplay between the accuracy (and resolution) of speed estimates, animal behaviour and VP inaccuracy could result in correction factors (see Discussion) that disproportionately (incorrectly) expanded sections of the dead-reckoned track (Fig. 7). For example, in Fig. 7, clear scaling errors arose between the third and fourth VP (post-VP correction) during soaring in a thermal when VeDBA- and GPS-derived speeds were used. At this path segment, the distance correction factor required when using constant speed values differentiated according to behaviour (green) was 3, juxtaposed with 22 and 47 for the VeDBA- (blue) and GPS- (red) derived speeds, respectively.

**Fig. 8:**
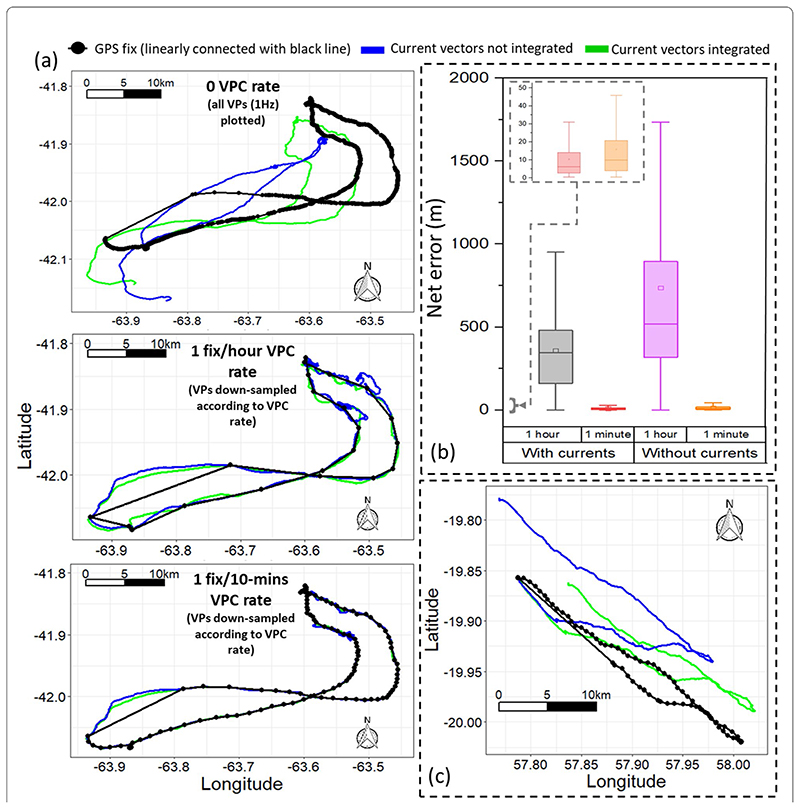
**a** One penguin’s dead-reckoned track calculated with- (green) and without- (blue) current integration and 3 variant VP correction rates (left panel). **b** Differences in net error when dead-reckoned tracks were iteratively integrated with space- and time-correction (net error estimates obtained from 5 penguin datasets). The boxes denote the median and 25–75% interquartile range and whiskers extend to 1.5*IQR. **c** An uncorrected dead-reckoned tropicbird flight path, relative to GPS, both with (green) and without (blue) current integration. Note the clustering of fixes (e.g., due to animal not moving much for extended periods of time) that can occur when using temporal sub-sampling routines (a more refined method could include using a VP correction rate of 1 fix every ‘x’ m moved (e.g., as estimated between VPs)).

The dead-reckoned tracks in air and water improved in general accuracy when suitably estimated external current flow vectors (tidal-/wind-speeds and direction per unit time and space) were incorporated (via travel vector and current flow vector addition—’current integration’) (Fig. 8).

## Discussion

### Speed inaccuracies and VP-corrected dead-reckoning

It is notable that the calculated examples of animals travelling on land (lions and walking penguins), had far less net error per given VP correction rate than the animals travelling in fluid media (swimming penguins, cormorants and tropicbirds), confirming the accuracy of the method for the former medium. However, dead-reckoning is very valuable for animals moving in fluid media (particularly for 3-D movement and movements underwater, which cannot be monitored by GPS), even though the inaccuracy is greatest at such times. There are three reasons for the greater inaccuracy:

1. The DBA approach of deriving speed estimates is temporally highly resolved and more accurate than GPS-derived estimates (used for tropicbirds) and the constant values used for part of the paths calculated for penguins and cormorants.
2. Typically, terrestrial species move slower than aerial/marine equivalents and thus incorporate less spatial error per unit time (cf. Additional file 1: Fig. S1).
3. External current flow vectors can cause the relationship between an animal’s (longitudinal axis) powered direction of travel and their true vector of travel to deviate [83, 84]. Indeed, in one of the earliest considerations of dead-reckoning for animals, Wilson et al. [33] noted that ocean currents were likely to be the greatest source of inaccuracy for positional fixes because of this.

Although convenient and powerful, DBA-derived speed has its own inaccuracies. The proposed linear relationship between DBA and mechanical power (cf. [63, 85]) presumably changes when the animal is load-bearing [86, 87], moving over a deformable substrate, over varying incline [88–90] or changing gait [64], or the attached logger undergoes motion independent of the body frame (e.g., collar roll), whilst even stationary behaviours can impart appreciable DBA, all of which may affect the relationship between DBA and speed [42, 90].

DBA-derived speed estimates can sometimes break down for species that hold appreciable quantities of air underwater (such as birds [65]) due to the compression of the air that takes place with increasing depth, with consequent changes in upthrust and power allocation according to swim angle [91] (cf. the difference of VeDBA magnitude between ascents and descents—Fig. 6). In addition, DBA does not scale reliably with speed for animals that glide, use thermals (cf. Figs. 5, 7), or bank and turn sharply [69, 92] because the more a gliding bird pitches down, the faster it will travel, even though there is no change in DBA. The same is true of animals with a higher density than water, such as elasmobranchs (cf. [93]), although in both cases the speed can be determined using the rate of change of altitude/depth if the pitch angle is known and it is high enough [48]).

Most previous studies that have used in-water speed sensors have done so by counting rotation of an external propeller or paddlewheel (e.g., [83, 94–97]). However, such systems have appreciable limitations with their ability to measure highly dynamic speed because {aside from environmental confounds such as blockage and turbulence (cf. [98])} flow characteristics around the sensor can change radically as a function of speed, most particularly in proximity to the animal body where the sensors are situated [99]. Recent research into fluid media speed sensors (e.g., [100]) though, may eventually provide systems that could markedly enhance the dead-reckoning process for animals travelling in water or air. Beyond this, the principal low-resolution methods for determining a speed proxy involve GPS-derived speed estimates or constant/simulated values according to behaviour-type or topological whereabouts (the primary approach used here for aquatic/aerial movement). Clearly, using constant speed estimates (even if they are a mode of the true value) quickly give erroneous integrated travel vectors, which emphasises the importance of appropriately spaced VP correction, particularly when speed is highly variable.

Generally, environmental covariate maps are typically given with lower resolution in aerial and aquatic domains, so location errors seem less important because spaceuse is, anyway, typically considered at larger scales (cf. [101]). For example, foraging ‘hotspots’ can be obtained based from 3-D dive profiles and even if dead-reckoning accuracy had an approximate 500 m error radius (e.g., 1 fix/hour VP correction rate for penguins (Fig. 1)), such errors seem more acceptable in an apparently predominantly featureless ocean (although not necessarily—e.g., investigating disturbances to animal movements created by underwater turbines). The same reasoning applies to most flying species although, because so many fly over land, higher absolute resolution is often required in order to map out the specifics of land-based features, such as wind turbines [102, 103] or thermals [104], that are relevant for bird (or bat) movement. Airflows themselves represent dynamic environments and assessing fine-scale dead-reckoned tracks in 3-D may reveal important interactions between animal and airscape and the energetic consequences involved (cf. [49, 72, 105, 106]). Most importantly, although dead-reckoning for fliers can incur substantial wind-based drift, GPS-based VP is usually accurate, because of the open sky which enhances signal transmission [13], and this can help correct tracks accordingly.

Whilst the accuracy of current flow vectors may be imprecise, their integration (see Gunner et al. [50] for method) can improve dead-reckoning estimates substantially (both pre- and post-VP correction; Fig. 8), which is especially important when VPs are scarce. It is worth noting however, that using GPS-derived speed and/or output from speed sensors estimating parameters of flow incorporates the speed of any current flow. Against this, assessing dead-reckoned travel vectors alongside VPs and external current flow vector estimates can provide insights into movement strategies of animals compensating for current drift (cf. [83, 84]).

The greater accuracy of VP-corrected dead-reckoning in terrestrial movement compared to fluid media is important because covariates of interest on land are typically highly resolved with, for example, habitat use [107, 108], conspecific interactions [109] and the effect of man-made structures [110, 111] (e.g., roads, fences, etc.) being of interest. Unlike most aquatic and aerial species, DBA can be continuously applied as the speed proxy for land animals, and the DBA–speed regressions (*m*- and *c*-values) can be modelled according to behaviour/terrain type, for higher resolution estimates [90]. In this, a primary factor in maximising dead-reckoning accuracy in a speed context, is to ensure that only periods of genuine traveling movement are dead-reckoned, since even stationary behaviours (e.g., grooming, feeding, rolling over, etc.) can impart appreciable DBA, which can inaccurately advance the vector of travel (cf. Fig. 3) [112]. Though notably, this is harder to achieve for animals in/on fluid media.

The VP correction procedure for distance, outlined in Walker et al. [41] and Gunner et al. [50] (and used here), divides the distance between consecutive VPs with the corresponding distance between temporally aligned dead-reckoned positions to obtain a distance correction factor (ratio) that is multiplied to all intermediate dead-reckoned distance moved estimates. This method has the advantage that the periods when the dead-reckoned vectors are not advanced (e.g., by allocating zero speed values for stationary behaviours, which can be determined from inertial data, e.g., [38]), are not subsequently expanded out in the linear drift correction procedure (since multiplying by zero achieves a zero-correction factor). Notably though, this method of correction can inflate error, beyond the normal linear vector expansion or contraction (cf. [113]). This is particularly problematic in small looping movements because if there is a disparity in the distance estimates between successive VPs and the corresponding dead-reckoned positions, path segments may be disproportionately expanded (and even inappropriately rotated) in order for the endpoints of both to align, even though such path segments may simply be an artefact of VP inaccuracy, heading error or (as demonstrated in Fig. 7), wrongly assigned speed values. This has consequences for space-use estimates and thus drives home the importance of initial behavioural identification, speed allocation, and VP screening prior to the VP correcting dead-reckoning procedure, particularly during highly tortuous movement. Erroneous estimates of speed can occur, for example, due to the DBA–speed relationship changing as a function of behaviour (i.e., thermal soaring), or using low-resolution GPS-derived speed. Specifically, in Fig. 7, this was because, respectively, the GPS frequency was not high enough to resolve the tortuosity of movement involved between fixes accurately (and thus the distance travelled, from which GPS-derived speed is calculated), and because birds typically impart negligible DBA during soaring behaviour. The latter effect, along with the presence of external wind currents, can substantially alter the required coefficients (gradient and intercept) and/or linearity of the DBA–speed relationship in time and space.

### Heading inaccuracies and VP-corrected dead-reckoning accuracy

Whilst not explicitly covered in these results, it is notable that because it is a vectorial operation, heading is the second major component in dictating dead-reckoning inaccuracy. Therefore, we clarify the potential causes of such error (first outlined in [50]) below. Heading is calculated using the arctangent of the ratio between two orthogonal components of the magnetic vector when the magnetic-field sensor is lying flat and parallel to the Earth’s frame of reference [114]. The tilt-compensated compass method rotates the attached tag’s magnetic vector coordinates and subsequently converts values of each magnetic vector channel to the corresponding Earth’s reference coordinate system, using the angles between the tag’s magnetic and the gravity vector. These angles are typically expressed as pitch and roll (Euler angles), which are resolved from the static component of acceleration (the gravity vector). The difficulty can be separating the static (due to gravity) and dynamic (due to the animal’s movement) components of acceleration (cf. [115]). Although various methods have been proposed to do this (e.g., using a running mean [59, 116] or high-pass filter [117]), estimates are problematic during periods of high centripetal acceleration (‘pulling *g’*; e.g., rapid cornering [92]), free-falling (no discernible or low gravity-component) [69] and highly dynamic movements [118]. Consequently, azimuth measurement error can be inflated at times when derived static acceleration estimates break down as a proxy of tag attitude relative to the Earth’s fixed reference frame.

Incorporating gyroscopes can improve the accuracy of computed heading, since they accurately reconstruct gravity-based attitude, irrespective of acceleration [119]. However, gyroscopes suffer from drift, high-power requirements and rapid memory consumption [120, 121]. Complex data processing makes them unappealing in most free-ranging bio-logging studies, particularly when information gains may be limited (cf. [122]). Further work should assess the extent to which gyroscopes do improve (species-specific) VP-corrected dead-reckoning accuracy, particularly at fine-scales (e.g., during fast, transient manoeuvres such as prey pursuit).

The usual method to derive Euler angles is to determine a set vectoral orientation with each orthogonal channel representing a particular body plane (anterior–posterior, medio-lateral and dorsal–ventral) with respect to the earth’s frame of reference [41, 60, 123], and the order of these channels is pivotal for deriving correct estimates of body rotation about the three axes [58] (for equations see; Gunner et al. [50]). However, this assumption breaks down for animals (or attached tags) that change orientations frequently at angles greater than perpendicular from their longitudinal and lateral axes of ‘normal’ posture due to the singularity issues (Gimbal lock) that arise when using the Euler sequence of 3-D vector rotation [124]. This problem can be mitigated by using a quaternion-based orientation filter [125, 126], however such an approach requires complex mathematical processing which may, in part, explain why Euler rotations are favoured (at least in bio-logging studies). We suggest that quaternion estimated heading should be compared with Euler angle-derived heading within the dead-reckoning framework, to assess the extent of error that occurs during times when the Euler sequence for determining attitude/orientation is likely to break down (e.g., during high centripetal acceleration). At the very least (when using Euler angles), inertial measurement coordinate frame adjustments of the tag frame (reflecting the body frame) relative to the Earth should be carried out [cf. 58] for animals that carry out ≥ 90° body inversions (e.g., a penguin walking *vs* swimming).

Small discrepancies between the tag and animal body coordinate frames are not as vital to correct for deriving heading since the tilt-compensated compass only concerns the attitude of the tag relative to the Earth so any required heading offset between the tag and animal’s body frame can be subsequently applied. In fact, consistent biases in tag heading are easily corrected for within the VP-corrected dead-reckoning framework, with the difference in heading from true North between consecutive VPs and corresponding dead-reckoned positions being applied as the heading correction factor (see [50] for method). However, there is no straight-forward solution to correcting heading from tags that move independently of the body (e.g., through partial dislodgment).

Animals that undertake long migrations can be subject to variations in the strength and declination of magnetic fields and this can be difficult to account for, because the magnetometry calibration procedure [127], required for correcting soft and hard iron distortions [128], is typically performed prior to deployment and is therefore only relevant according to the specific magnetic conditions of that area. Even after sufficiently calibrating magnetometry data, local changes in the magnetic field (e.g., due to the presence of ferrous material) and temperature-induced offsets [58, 129] can introduce channel bias in measured magnetism, confounding heading output. Moreover, the horizontal components of the magnetic field become small when the magnetic-field inclination angle increase towards the poles, which can also result in heading measurement error [58]. Lastly, heading estimates assume the animal moves in the direction of its longitudinal axis, which is not always the case [67].

### VP inaccuracies and VP–corrected dead-reckoning accuracy

Data collected from tags attached to neck collars generally show more variation in acceleration and magnetic-field intensity values than data obtained from loggers deployed near an animal’s Centre of Mass (CoM). This is because collars can roll independently to that of the body frame. That our net error estimates plateaued for (collared) lions at *ca*. 10 m, with a 1 fix/30 min VP correction rate demonstrates though, the value that VP-corrected dead-reckoning can have for constructing long-term, fine-scale terrestrial movement. Indeed, across all VP correction rates, distance moved estimates alone were more consistent (and higher) when estimated between dead-reckoned positions than VPs (Fig. 2). The sharp increase that occurs in distance moved estimates (at the highest VP correction rate) stems principally from incorporating all the VP locational error (Fig. 2). Notably, the temporal sub-sampling intervals of VP correction were not always exact because fix success can fail for periods longer than the set VP correction rate (e.g., during submersion in water) [130]. As such, we advocate that the VP correction rate should not be treated literally between species with the number and regularity of VP correction generally lower for aquatic animals per set VP sampling rate. Indeed, dead-reckoned distance moved estimates were generally much higher than the equivalent VP distance in aquatic and flying species. This is because VPs can fail for extended periods while dead-reckoning is continuous.

It is worth reemphasising that across all travel media, dead-reckoning accuracy as assessed via net error must not be taken literally (particularly at high VP correction rates), since VP error can also be appreciable (cf. Fig. 4, Additional file 1: Fig. S2), whilst net error does not account for inaccuracies between VPs (cf. Fig. 7) and extremely high values at single points in time (likely due to VP error) may increase overall net error estimates (cf. Fig. 3). Only including fixes where genuine travelling movement occurred (e.g., as assessed from motion sensor data) can help remove GPS error that occurs when animals are stationary or extremely slow-moving (e.g., tortoises) where the disparity between VP error and genuine travelling movement become disentangled (even at low VP correction rates).

### Deciding drift correction rates

The specific number of VPs that are required to drift correct are obviously species-specific and there are many confounds to this process that we outline above, including user-defined track-scaling and initial VP screening, that will change on a case-by-case basis. The scenarios outlined above should provide a general idea of the required correction rates for the resolution that is required in aerial, aquatic and terrestrial domains. In essence, we suggest that VP correction should be undertaken as little as possible, but as much that is required. For investigating highly defined scales of movement (for example here, lion–fence boundary interactions or penguin navigation strategies on land) then 1 fix/15 min or more may be required—particularly during highly dynamic and tortuous movements when net error is generally greatest (cf. Additional file 1: Fig. S2) and when speed estimates may be unreliable (cf. Fig. 7). For longer-term studies (e.g., weeks to months) general movement networks and distance moved estimates, where net errors of *ca*. 200 m, may be deemed reasonable definition for the questions being asked, much lower VP correction rates could be used to preserve battery life, allowing animals to carry smaller tags. Importantly, even when high VP correction rate is possible (e.g., ≥ 0.1 Hz), corrections should only be carried out at times of genuine traveling movement, whereby distance moved between VPs exceeds the positional error radius stemming from the precision of their measurement.

### The utility of dead-reckoning

The vast majority of animal tag studies investigating space-use have done so subject to the resolution of the VP system utilised (typically GPS), something that has generally resulted in low-aspect ratio location-based point density (cf. [131]) or diffusive straight-line movements (cf. [3]). VP-corrected dead-reckoning provides a means to incorporate all the various scales and directions of movement between VPs (rather than just linear interpolation [14]) and thus has the capacity to map out movement patterns to a hitherto-unrealised degree [46]. Such expansion of the resolution of animal space-use into fine-scale, uninterrupted movement path networks can enhance insight into a number of fundamental concepts considered important in structuring movement paths and space-use by animals, including energy landscapes [132], landscapes of fear [133] and accident landscapes [134]. VP-corrected dead-reckoning has particular relevance for marine underwater studies because 3-D movement can be reconstructed [54, 67] at times when VPs cannot be obtained [130] (e.g., Fig. 6).

The immediate benefits of using VP-corrected dead-reckoning are:

1. That it can reconstruct continuous, fine-scale 2-/3-D movement paths, irrespective of the environment and at higher resolution than any VP system [42, 50]
2. That it provides a means to reduce the recording frequency of GPS locations, thus extending battery life and/or reducing deployment bulk/weight [46]
3. That it prevents/limits positional noise (‘jitter’) of ‘high-res’ (e.g., ≥ 1 Hz) GPS datasets, which is most apparent during non-moving behaviours such as rest and in highly heterogenous environments where radio signal can be easily obstructed (cf. [22, 23])

In particular, the scales of tortuosity exhibited between VPs, as defined with VP-corrected dead-reckoning, irrespective of the drift from true location (net error), can highlight behaviours that VPs alone cannot. For example, we demonstrate here that circling behaviour [53] can easily be distinguished in dead-reckoned tracks from tropicbirds (Fig. 5), even when the circling duration is as low as 10 s. VP-corrected dead-reckoning can also greatly improve the accuracy of space-use estimates by limiting the inclusion of positional noise via advancing travel vectors and carrying out VP correction only at times when the animal is determined to be travelling [112]. In fact, we believe that a particular value of VP-corrected dead-reckoning, is that it will provide important detail about the effects of humans and anthropogenic landscape features on animal movements, a topic that is increasingly germane [135–137]. For example, understanding the extent of the permeability of anthropogenic barriers (e.g., fences, roads) and the hazards that they pose to specific animals [138–140] is key to proper livestock and wildlife management [141–144]. Our work demonstrates that this approach details the intricacies of animal–barrier interactions, including the locations of barrier transgression as well as movement paths pre-, during and post-barrier transgression. Moreover, VP-corrected dead-reckoning should also elucidate animal foraging and predator avoidance strategies as well as provide vital information that will help us understand how animals respond to, and navigate through (air/tidal) current flows [84]. Beyond this, dead-reckoning has been demonstrated to have high welfare value in zoos, by enabling continuous assessments of enclosure space-use relative to enrichment regimes and the possible occurrence of stereotypical behaviours such as pacing [112].

Importantly, this approach has implications for informing conservation management. For instance, the impacts of free-ranging forest elephants depend largely on what they are doing at very specific localities [145, 146]. At present, GPS is mostly used to reflect on where elephants move as a general response to the availability of resources such as food, water and safety (e.g., [145, 147, 148]). Drift-corrected dead-reckoning can highlight the specifics of behaviours and localities, and therefore, for example, allow researchers to retrace elephant movements to determine what elephants feed on and where they do it, which has obvious management value. Lastly, alongside capturing underwater movements, dead-reckoning may prove effective for elucidating movement-specific behaviours in other habitats that have poor signal reception, such as within caves and burrows.

### Key considerations governing the relationship between VP correction rate and dead-reckoning accuracy

To improve VP-corrected dead-reckoning estimates (assuming the accelerometer–magnetometer Euler angle approach), the minimum pre-routine should consider the following:

1) Screening for, and removal of, erroneous VP estimates.
2) A suitable magnetometer calibration [127, 149] with correction of acceleration and magnetometry data for any discrepancies between the tag coordinate frame and body coordinate frame, relative to the Earth’s fixed frame of reference (e.g., by visually taking note of the deployment angle offset and derotating using rotation matrices as outlined in [58]).
3) Application of any required magnetic declination offsets (and approximate yaw offset if step 2 was not carried out).
4) Computation of suitable estimates of speed (possibly modulated according to identified behaviour and/or terrain type).
5) Integration of external current flow vectors where appropriate (and when reasonably modelled/measured).
6) Post-examination of dead-reckoned tracks (both pre- and post-VP correction), relative to VPs, visually to examine and readjust aspects of the initial track-scaling.

Further advances could include additional limb-borne logger deployments that may decipher limb stride frequency via clearer stylised patterns of inertial measurement [39, 150, 151]. Such counts per unit time, may themselves be used as a speed proxy [50]. Whilst not covered here, investigation of extremely high or biased distance (speed) and heading correction factors may be used to aid in identifying inaccuracies originating from tag performance (heading, speed and/or VP inaccuracy) [50]. Very low distance correction factors (< 1) either indicate inaccurately identified bouts of travelling movement or supplying inaccurately high-speed estimates. On the other hand, very high distance correction factors (> 1) again, could indicate inaccurately identified bouts of travelling movement, or supplying inaccurately lowspeed estimates or, the most likely cause is due to VP error. Consistency in the direction of heading correction factors either indicate a yaw offset of the tag relative to the animal’s coordinate frame, a hard iron offset in magnetic data (or a required summation of the magnetic declination), or due to external current flow drift.

**Fig. 9:**
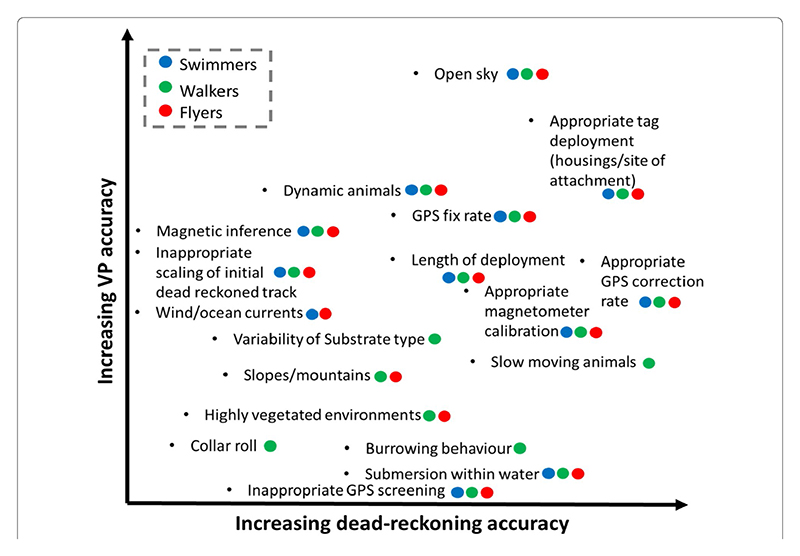
Schematic diagram to illustrate the various elements that modulate VP and VP-corrected dead-reckoning accuracy. Black dots illustrate the element’s graphical position

Generally, the factors that affect dead-reckoning and VP accuracy are illustrated in Fig. 9, with the level of obtainable dead-reckoning accuracy depending on the user-defined initial track-scaling, VP screening and the study species.

## Conclusion

Combining dead-reckoning and VPs (specifically, GPS) produces an extraordinarily powerful method for looking at animal movement. Under ideal conditions, VP-corrected dead-reckoning can enhance the resolution of animal movement from diffusive area-use to high-resolution animal pathways. We have highlighted the main sources of inaccuracy within the dead-reckoning framework and considered the implications of such error across a diverse group of animals using different modes of movement and operating in the three main media. A major improvement to this approach necessitates accurate speed estimates (particularly in fluid media). Further work could build on these fundamentals and investigate the utility of VP-corrected dead-reckoning across a suite of animals and environments. Appropriate sharing of finding would provide a repository of species-specific rules for assessing movement-specific behaviours, VP inaccuracy, speed allocation and heading computation for the community to benefit from maximum resolution of animal movement.

## Supplementary Material

Supplementary material

## Figures and Tables

**Table 1:** Experimental protocol for each species

Animal (number (n) assessed)	Scheduled GPS frequency	DR frequency	Approx. DR length	Extrapolated speeds	Capture and deployment method
Lion (n = 10)	1 Hz	1 Hz	2 weeks	DBA-based speed Due to high variability in step gaits, m- and c-values were computed per individual from VeDBA ~ GPS-derived speed regression (cf. [23])	Prides were lured to bait using audio recordings and individuals were anaesthetised at night according to SANParks operational proce- dures—detailed in SANParks’ ‘Standard Operating Procedures for the Capture, Transportation and Maintenance in Holding Facilities of Wildlife’ Units were mounted to a Litetrack collar [https://www.lotek.com]. Collars were loose enough to allow three fingers to pass through
Penguin on land (walking) (n = 15 – 2 DR paths per individual (out- and inbound)	1 Hz	10 Hz	30 min	DBA-based speed Due to having a constant step gait, m- values were selected (c = 0) per individual based on the best scaling relative to GPS path pre-VP correction	Penguins were caught at the nest during the chick rearing season using the clipboard method [74] and cormorants were caught at the nest during the chick rearing season via a crook on the end of a long pole (cf. [75]). Birds were blind folded and restrained on a researcher’s knees Devices fitted longitudinally to the base of the spine using Tesa^®^ tape [74, 76, 77]
Penguin at sea (diving) (n = 15)	1 Hz	2 Hz	1.5 days	Change between constant values (according to behaviour-type) and vertical movement-based speed speed = 0.416 m/s (cf. [78]) when depth ≤ 0.3 m (cf. [79]) speed = 2.1 m/s (cf. [80, 81]) when depth > 0.3 m and absolute values of pitch were < 10° speed = Δd/tan(θ o π/180) (upper cap of speed derived this way = 3 m/s) when depth > 0.3 m and absolute values of pitch were ≥ 10°	
Cormorant at sea (flying and diving) (n = 15)	1 Hz	10 Hz	8 h	Change between constant values (according to behaviour-type) and vertical movement-based speed speed = 12 m/s when flying (derived from the heave acceleration (cf. [69]) speed = 0.1 m/s when resting at the sea surface (derived from depth sensor and lack of dynamic acceleration) speed = Δ*d*/tan(θ o π/180) (upper cap of speed derived this way = 3 m/s) during the ascents and descents of dives speed = 0.4 m/s during the bottom phase of dives	
Tropicbird at sea (flying) (n = 7)	1 fix every minute	10 Hz	3 h	GPS-based speed speed = Haversine distance between GPS fixes divided by the time period between values and linearly interpolated (cf. [82]). Speed values overwritten as 0.1 m/s when birds were resting at sea surface	Devices were placed in a zip-lock bag, inside unheated heat shrink wrap and fixed longitudinally to the back feathers [72] using Tesa^®^ tape [76]

‘Δ*d*/tan(*θ* · *π*/180)’ refers to the rate change of depth (m/s) divided by the tangent of the body pitch (converted from degrees to radians). DR = dead-reckoning and DBA = dynamic body acceleration. m- and c-values represent the (multiplicative) coefficient (gradient) and constant (intercept) of the VeDBA–speed regression [speed = VeDBA · *m* + *c*]

## Data Availability

All dead-reckoning protocols (and assessment of net error and distance moved) used in this study (including the *Gundog.Tracks* function) are supplied as complimentary R files, uploaded to GitHub [152]. Online scripts will be continually updated and any queries, suggestions and/or reported bugs should be emailed to the corresponding author.
