# Supplementary material for: How often should dead-reckoned animal movement paths be corrected for drift?
Source: Anim Biotelemetry. Author manuscript; Available in PMC 2021 Dec 10. (PMC7612089; doi:10.1186/s40317-021-00265-9)
Supplement: Supplementary material [file EMS140359-supplement-Supplementary_material.docx]

**
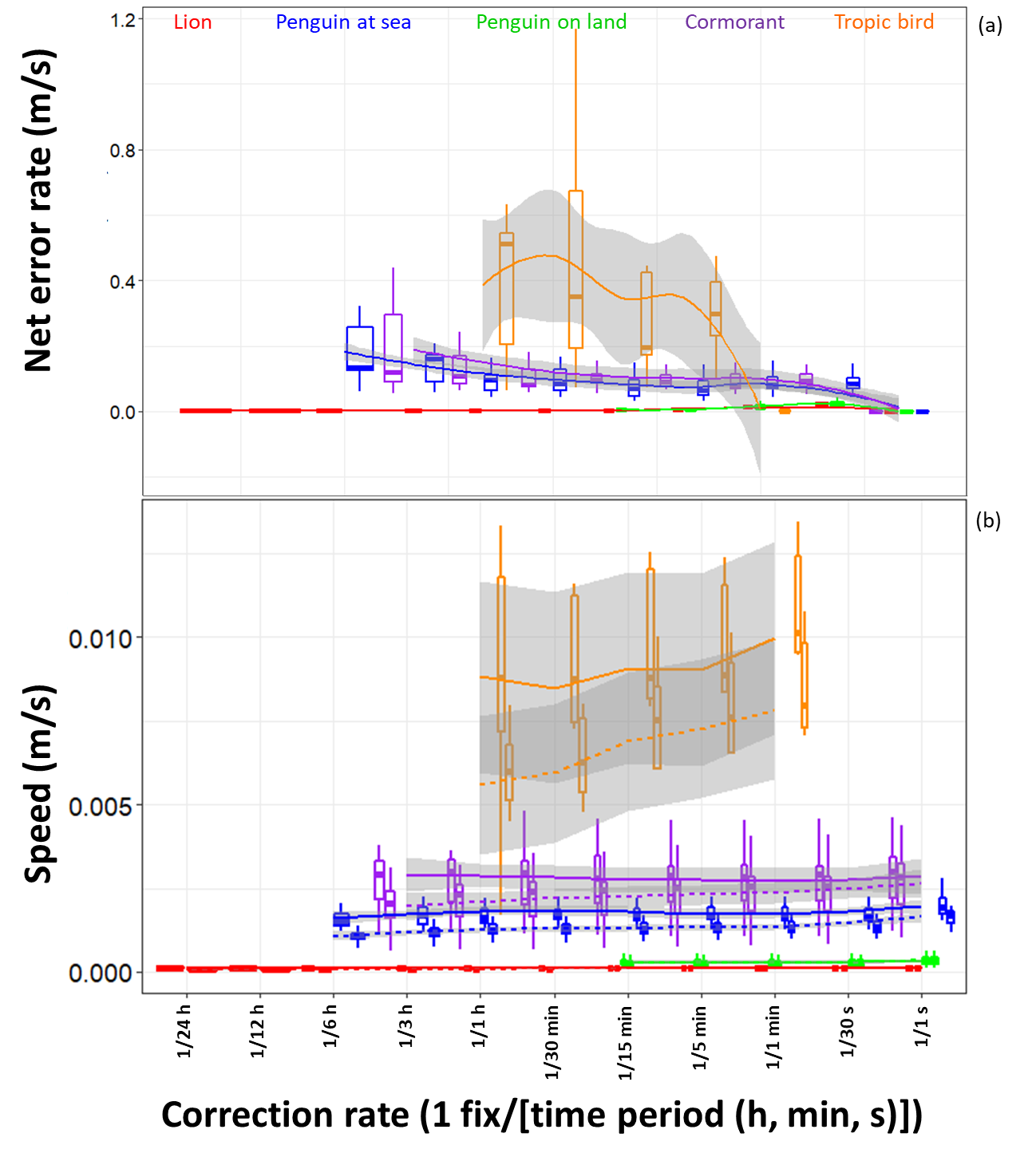
Supplementary Information**

Figure S1. Boxplots demonstrating the magnitude of net error (top panel) and distance moved (bottom panel), per VP correction rate and animal. Net error (a) is standardised according to the mean time between corrections (m/s) and distance moved (b) is standardised according to trip duration, respectively, per individual and VP correction rate to provide an approximate rate in m/s. Mean values were aggregated per individual and VP correction rate. Boxes encompass the 25-75 % interquartile range and horizontal bars denote the median value with ‘loess’ smooth line (grey shading show the standard error and Whiskers extend to 1.5 * Interquartile range). Note net error drops to zero when the VP correction rate equates with GPS recording frequency (1 Hz for the lions, penguins and cormorants, and 1 fix/min for the tropicbirds).


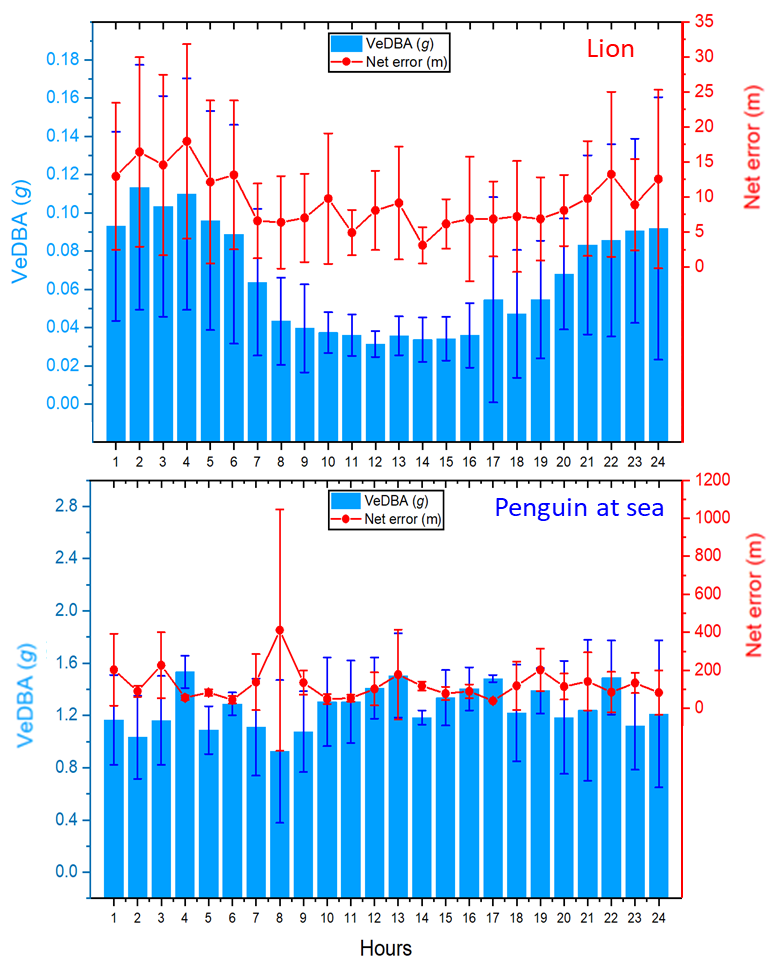


Figure S2. Mean (± 1 SD) VeDBA (column/bar) and net error (line & symbol) per hour over the duration of three individual lion (top panel) and three penguin (bottom panel) dead-reckoned tracks, VP corrected approx. every 30 minutes. Note that the net error of lions was generally slightly higher and more variable during the night when they were more active, although net error was still appreciable during the day when lions were predominantly resting, principally due to VP error. On the other hand, penguins demonstrated no clear circadian trends in activity or net error, in part due to the high variability between the dynamism of recorded movement and speed of travel (e.g., due to preening behaviour during ‘surface rest’ periods and the constant presence of external current flow vectors), as well as the variability in the time of day (and duration) of rests. Note that the y-scales differ between the species.

**Text S1. Dead-reckoning formulae**

1. **Compute the distance coefficient (**$\boldsymbol{q}$**)**

$q=\frac{s \bullet TD}{R}$

where $s$ refers to speed (units in m/s), $TD$ refers to the time difference between values (units in s) and $R$ is the approximate radius of the earth (R = 6378137 m).

1. **Derive longitude and latitude coordinates**

${Lat}_{i}=asin\left( \sin{(Lat}_{0})\bullet\cos(q)+\cos{(Lat}_{0})\bullet\sin(q)\bullet\cos(h) \right)$

${Lon}_{i}={Lon}_{0}+atan2\left( \left( \sin(h)\bullet\sin(q)\bullet\cos{(Lat}_{0}) \right), \left( \cos(q)- \sin({Lat}_{0})\bullet\sin({Lat}_{i} \right)) \right)$

where ${Lat}_{0}$,${Lat}_{i}$ and ${Lon}_{0}$,${Lon}_{i}$ are the previous and present latitude and longitude coordinates, respectively, $h$ is the heading and$q$ is the distance coefficient. Note that the coordinates and heading must be supplied in radians.

1. **VP-correct procedure**

The distance ($d$) and bearing (b) between consecutive VPs (used to correct) and consecutive (time-matched) dead-reckoned positions are calculated based on the below formulae;

$$d={2\bullet R\bullet sin}^{-1}\left( \sqrt{{sin}^{2}\left( \frac{{Lat}_{i}-{Lat}_{0}}{2} \right)+\cos\left( {Lat}_{0} \right)\bullet cos\left( {Lat}_{i} \right)\bullet{sin}^{2}\left( \frac{{Lon}_{i}-{Lon}_{0}}{2} \right)} \right)$$

where R is the Earth’s radius and the output $d$ is in metres. Note this is the Haversine formula.

$$b=atan2\left( \begin{aligned} sin\left( {Lon}_{i}-{Lon}_{0} \right) \bullet\cos\left( {Lat}_{i} \right), \\ cos\left( {Lat}_{0} \right) \bullet sin\left( {Lat}_{i}-{Lat}_{0} \right) \bullet cos\left( {Lat}_{i} \right) \bullet cos\left( {Lon}_{i}-{Lon}_{0} \right) \end{aligned} \right)\bullet\frac{180}{\pi}$$

where $b$ output is in the scale -180 ^o^ to +180^o^. To convert $b$ to the conventional 0^o^ to 360^o^ scale, 360 should be added to values < 0.

The distance between each VP is divided by the distance between the corresponding dead-reckoned positions to provide a distance correction factor;

${Distance}_{corr.factor}= \frac{{Distance}_{VP}}{{Distance}_{DR}}$

The bearing (or rather, heading) between each VP is subtracted by the bearing between the corresponding dead-reckoned positions to provide a heading correction factor;

${Heading}_{corr.factor}= {Bearing}_{VP}- {Bearing}_{DR}$

To maintain a maximum potential difference of 180^o^ in either circular direction, 360 should be added to values < -180 and 360 subtracted from values > 180. All intermediate $q$ values are multiplied by the distance correction factor and the heading correction factor is added to all intermediate $h$ values (ensuring that $h$ values are in degrees). To ensure circular range remains between 0^o^ and 360^o^ for the updated $h$ values, 360 should be subtracted from values > 360 and added to values < 0. Note, these corrections for ensuring that circular range is maintained (adding/subtracting 360^o^) when deriving and applying the heading correction factors are not required if both $b$ and $h$ are in radians. It can be more intuitive though to have units in degrees when assessing biases in motion sensor derived heading offset/error, relative to VPs. After the correction factors have been applied, step 2 is repeated. Occasionally more than one iteration of the formulae (steps 2 and 3) is required for the path to adhere ‘exactly’ with the ground-truthed locations.
